# Comparison of the Transmembrane Mucins MUC1 and MUC16 in Epithelial Barrier Function

**DOI:** 10.1371/journal.pone.0100393

**Published:** 2014-06-26

**Authors:** Ilene K. Gipson, Sandra Spurr-Michaud, Ann Tisdale, Balaraj B. Menon

**Affiliations:** Schepens Eye Research Institute of Massachusetts Eye and Ear, Department of Ophthalmology, Harvard Medical School, Boston, Massachusetts, United States of America; University of Nebraska Medical Center, United States of America

## Abstract

Membrane-anchored mucins are present in the apical surface glycocalyx of mucosal epithelial cells, each mucosal epithelium having at least two of the mucins. The mucins have been ascribed barrier functions, but direct comparisons of their functions within the same epithelium have not been done. In an epithelial cell line that expresses the membrane-anchored mucins, MUC1 and MUC16, the mucins were independently and stably knocked down using shRNA. Barrier functions tested included dye penetrance, bacterial adherence and invasion, transepithelial resistance, tight junction formation, and apical surface size. Knockdown of MUC16 decreased all barrier functions tested, causing increased dye penetrance and bacterial invasion, decreased transepithelial resistance, surprisingly, disruption of tight junctions, and greater apical surface cell area. Knockdown of MUC1 did not decrease barrier function, in fact, barrier to dye penetrance and bacterial invasion increased significantly. These data suggest that barrier functions of membrane-anchored mucins vary in the context of other membrane mucins, and MUC16 provides a major barrier when present.

## Introduction

**Figure 1:**
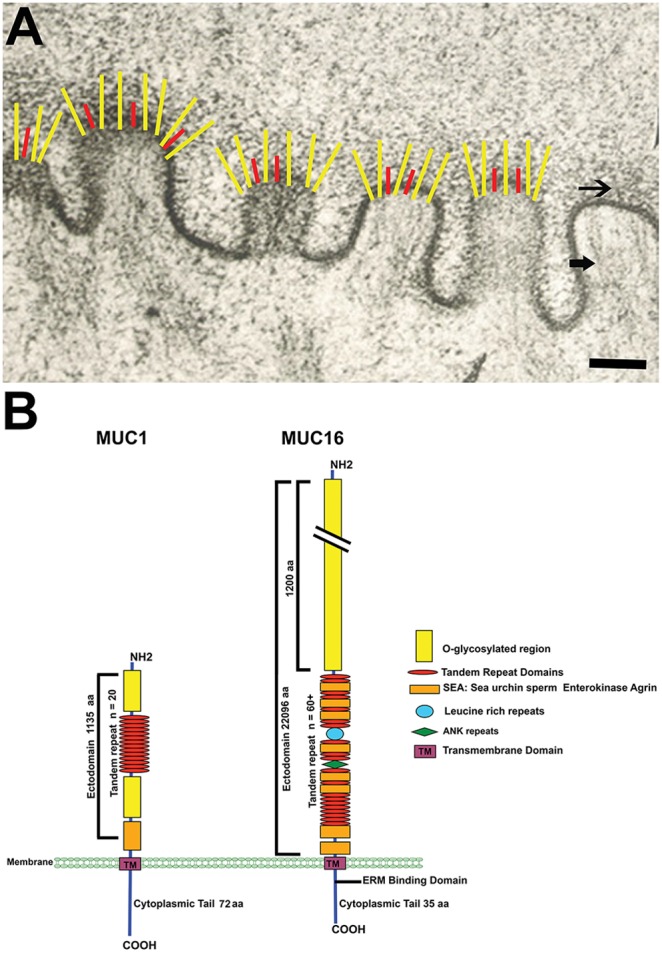
Diagram of the distribution of the MAMs MUC1 and MUC16 in the epithelial glycocalyx and their molecular domains. (A) Electron micrograph showing diagrammatically, the distribution of MUC1 (red) and MUC16 (yellow) within the electron dense glycocalyx (top arrow) present at the tips of membrane folds or microplicae of an epithelial cell. Note the actin filaments inserting into the membrane at the tips of the microplicae where the cytoplasmic tails of the membrane mucins are present (bottom arrow). (B) Both MUC1 and MUC16 have a short cytoplasmic tail, a transmembrane domain and an extended, highly glycosylated extracellular domain that contains tandem repeats of amino acids, rich in serine and threonine, that allow the heavy O-glycosyation of the molecules. MUC1 has one sea urchin sperm protein, enterokinase and agrin (SEA) module, whereas MUC16 has multiple SEA modules interspersed within tandem repeats and, in addition, a shorter cytoplasmic tail and an ERM binding domain. Note that the MUC16 ectodomain is approximately 20 times longer than that of MUC1. It has been estimated that MUC16 can extend up to 250–300 nm into the glycocalyx [43]. (Electron micrograph taken from [50] with permission.) Scale Bar = 500 nm.

The apical glycocalyx of epithelia of mucosae lies at the interface between the external environment and the mucosal tissue. As such, it provides a protective barrier that prevents pathogen adherence and internalization as well as a selective barrier to penetrance by other compounds. Major components of the glycocalyx are membrane-anchored mucins that are also termed membrane-spanning, membrane-bound or membrane-tethered mucins (Fig. 1A) (for review see [1], [2], [3]).

Mucins are heavily O-glycosylated glycoproteins that share the feature of tandem repeats of amino acids within their protein backbone, these repeats are rich in serine and threonine, providing sites for the association of O-glycans. Two types of mucins have been identified–secreted and membrane-anchored (MAMs). Unlike the secreted mucins that are produced by epithelial goblet cells and mucosal glands, MAMs lack N- and C-terminal region cysteine-rich domains that allow homomultimerization to form thick mucus, and have instead, a membrane-spanning domain and a short cytoplasmic tail that tethers the mucin to the apical surface. All wet-surfaced mucosal epithelia express MAMs including those of the ocular surface, and respiratory, gastrointestinal and genitourinary tracts. Mucins have been named in order of discovery MUC 1, 2 etc., with “MUC” designating human genes, and “Muc” mouse genes. The membrane-anchored mucins include MUC1, MUC3A, MUC3B, MUC4, MUC12, MUC13, MUC15, MUC16, MUC17, MUC20, and MUC21, with MUC1 being ubiquitously expressed and MUC16 the largest of the group. The repertoire of MAMs in regions of wet-surfaced mucosae varies. For example, MUCs 1 and 16 are expressed by epithelia of the ocular surface, and respiratory and female reproductive tracts, whereas MUCs 3, 12 and 13 are predominant on gut epithelial surfaces (for review see [1], [2], [3], [4], [5]).

Several of the MAMs have been reported to be multifunctional, having both surface barrier functions and documented signaling functions either through their cytoplasmic tails or through EGF-like domains located near the membrane-spanning region in the ectodomain [2], [3]. The most studied of the MAMs have been MUCs 1, 4 and 16, particularly as each are tumor cell markers and are highly upregulated in breast, pancreatic and ovarian cancers, respectively (for review see [1]). As a result of their association with cancers, the majority of studies of their functions have been documented in cancer cell lines, whereas understanding the functions of specific MAMs in the glycocalyx of native mucosal surfaces has lagged. In those studies of the function of MAMs in native epithelia that have been done, the ectodomains, particularly of MUC1 and MUC16 (also known as the CA125 antigen), are ascribed similar functions, that of preventing adherence/penetrance of pathogens and cell-cell adhesion [6], [7]. A comparison of the molecular structure and size of MUC1 and MUC16 (Fig. 1B) demonstrates that, of the two mucins, the ectodomain of MUC16 is about 20 times larger than that of MUC1 and its ectodomain includes a number of sea urchin sperm protein, enterokinase and agrin (SEA) modules, whereas MUC1 has one SEA module [7]. These modules are found in many membrane-associated proteins that are released from the cell surface [8].

As examples of MUC1’s reported role in pathogen barrier function, adenoviral penetrance into airway tracheal bronchial epithelia is increased in mice null for Muc1 [9]. Additionally, Muc1 limited *Helicobacter pylori* binding to gastric epithelial cells, and expression of MUC1 enhanced resistance to *C. jejuni* cytolethal distending toxin (CDT) *in vitro* and in CDT null mice, *C. jejuni* showed lower gastric colonization in Muc1(−/−) mice in vivo [10]. Since the sequence and ectodomain sizes of human and mouse MUC1 and MUC16 vary greatly (BLAST database comparisons) and since the mucosal epithelial expression profiles of MUC16 varies greatly between humans and mice [11], it is difficult to draw conclusions regarding the function of human mucin genes from Muc null mice. Thus studies of human mucin genes have employed in vitro models, showing for example, over-expression of MUC1 has been demonstrated to prevent E-cadherin mediated cell-cell adhesion [12]. MUC16, the largest of the MAMs, with an extracellular domain of approximately 22,000 amino acids, has been demonstrated to be a barrier to bacterial adherence [13] and internalization [14] as well as to penetrance of dyes [13], [15]. MUC16 also has anti-adhesive properties in that this MAM has been demonstrated to prevent adherence of trophoblast cells to uterine epithelia [5].

Studies testing the roles of the MAMs in barrier function of native epithelia have studied only one mucin per epithelium, despite the fact that most epithelia express and place several mucins at their apical surfaces. There is no information on the relative roles of MAMs in barrier function within the same epithelial glycocalyx. The purpose of the study reported herein, was to compare the barrier functions of MUC1 and MUC16 in the same mucosal epithelial cell type. The human corneal epithelium has only two of the large membrane mucins in its apical glycocalyx and thus represents an excellent model for comparison of the barrier function of these two mucins. Results reported here demonstrate distinct differences between MUC1 and MUC16 barrier function, ability to prevent dye penetrance and bacterial adherence/internalization. Surprisingly, the comparison also demonstrated that MUC16 exhibits additional barrier function in that it contributes to tight junction formation, transepithelial electrical resistance (TER) and to apical epithelial cell surface area, whereas MUC1 does not.

## Materials and Methods

### Ethics statement

As described previously for development and characterization of the telomerase transformed human corneal epithelial cell line (HCLE) used in this study [16] human corneal epithelial cells were derived from human corneoscleral rims provided by Roger Steinert and Ann Bajart of Ophthalmic Consultants of Boston. For comparing amount of MUC16 antibody binding on apical surfaces of epithelial cells in culture to that of native tissue discarded full thickness human corneal epithelial sheets, removed by epikeratome for corrective refractive surgery, were kindly provided by Ula Jurkunas, MD, of the Massachusetts Eye and Ear Infirmary. These samples were obtained without patient identifiers as discarded tissue post surgery and the Schepens Eye Research Institute Institutional Review Board (IRB) waived the need for approval and consent.

### Generation of stable MUC knockdown cells and cell culture

MUC1 was stably knocked down in the previously described telomerase transformed human corneal-limbal epithelial (HCLE) cell line [16] by two rounds of transfection with 1 µg of the plasmid psiRNA-H1b-MUC1 (InVivoGen) encoding a hairpin, targeting the MUC1 gene, using Polyfect transfection reagent (Qiagen) in the first round, followed by Effectene transfection reagent (Qiagen) in the second round to improve the transfection efficiency. The siMUC1 sequence used (5′-ACCTCCAGTTTAATTCCTC-3′) was previously reported to be efficient for knockdown of MUC1 in pancreatic tumor cells [17]. Stable transfectants were selected with 5-µg/ml blasticidin and the resultant cell line was designated shMUC1 knockdown cells. Control cells were similarly generated by transfection with a plasmid containing a nonsense, scrambled siRNA (psiRNA-hH1blasti-LUC; InVivoGen) or were non-transfected cells. These cell lines were designated scr1 or NT cells, respectively. Stable knockdown of MUC16 in HCLE cells using pSuperRetro-puro containing MUC16 siRNA sequence 2 (5′-CTGCATCTACTCCCATCTC-3′) was previously reported [13] and cells were designated as shMUC16 cells. Controls were similarly generated using nonsense, scrambled siRNA and were designated as scr16 cells.

Non-transfected (NT), scrambled shRNA transfected (scr1, scr16) and shMUC1 or shMUC16 transfected HCLE cells were grown in keratinocyte serum-free medium (Invitrogen) supplemented with 25 µg/ml bovine pituitary extract, 0.2 ng/ml epidermal growth factor and 0.4 mM CaCl_2_ to confluence, followed by DMEM/F12 plus 10% calf serum and 10 ng/ml EGF for 7 d to achieve optimal mucin production [16]. RNA was harvested using TRIzol reagent (Invitrogen), and protein with 2% SDS plus protease inhibitors (Roche Diagnostics). All experiments were performed a minimum of two times with a minimum of 3 replicates per experiment.

### Quantitation of mucin protein

MUC1 and -16 proteins in cell lysates or present on the cell surface (isolated by capture of biotinylated cell surface proteins using the Pierce Pinpoint Cell Surface Protein Isolation Kit (ThermoScientific) following the manufacturer’s recommendations) [18], [19] were separated on 1% SDS-Agarose gels [20], [21], transferred to nitrocellulose and assayed by Western blot [22], using antibodies 214D4 (Upstate) to MUC1 and anti-human CA125, Clone M11 (NeoMarkers) to MUC16. Blots were reprobed with antibody to GAPDH (Santa Cruz Biotechnology) as a loading control. Densitometric analyses of protein bands recognized by MUC1 or MUC16 antibodies were performed using 1D Image Analysis Software, Version 2.02 (Eastman Kodak, Co.). Data for total cellular MUC were expressed as MUC normalized to GAPDH and for cell surface MUC normalized per equivalent cm^2^ of growth area and then both expressed relative to HCLE NT cells.

### Assessment of barrier function

#### Rose bengal dye penetrance

shMUC1, shMUC16 cell lines, and NT, scr1, and scr16 control HCLE cell lines grown for optimal mucin production, were rinsed with PBS and incubated for 5 min with 0.1% solution of the anionic dye rose bengal in Ca^2+/^Mg^2+^-free PBS. Dye was aspirated, 5 images per well were immediately photographed at room temperature (RT) with a 10X objective on a Nikon Inverted Eclipse TS100 microscope with a Spot Insight camera (Diagnostic Instruments, Inc.), and areas excluding the dye, representing the areas protected from dye penetrance, were quantitated using ImageJ software (NIH) as previously described [13], [14], [15].

#### Bacterial adherence and invasion

Epithelial cells were grown for optimal mucin production, antibiotics were removed from the culture medium for the last 24 h of culture, and cells were rinsed with unsupplemented DMEM/F12 prior to addition of bacteria. Following incubation with bacteria, the cultures were rinsed 3 times with sterile PBS before proceeding to the assays for adherence and invasion.

Two methods were used to assess bacterial adherence. Epithelial cultures were A) incubated with 2×10^7^ colony forming units (cfu) of FITC-labeled *Staphylococcus aureus,* strain ALC1435 [23], which had been labeled for 30 min on ice with 0.1 mg/ml of FITC in PBS, collected by centrifugation and washed 6 times with PBS prior to re-suspension in DMEM/F12 for 1 h. The number of adherent bacteria per microscopic field were quantitated using Image J [13] or B) incubated with 5×10^7^ cfu of the *Staphylococcus aureus* for 1 h, and bacteria adherent to cells were determined by plating aliquots of serial dilutions of epithelial cells on agar plates as previously described [24]. Bacterial invasion was assayed following incubation with 5×10^7^ cfu *Staphylococcus aureus* for 4 h, treatment with gentamycin and penicillin to kill extracellular bound bacteria and plating of serial dilutions of epithelial cells lysed with 1% saponin [14], [25]. In the last two assays, the number of recovered bacteria was expressed as a percentage of bacteria initially added to the cultures.

#### Tight junction function

Tight junction function was assayed by measuring transepithelial electrical resistance (TER) using an EVOM^2^ Epithelial Voltohmmeter (World Precision Instruments) [26], [27] in shMUC1, shMUC16 cells, as well as NT, scr1, and scr16 control cells that were plated on 0.4-µm pore Transwell inserts (Corning) and grown for optimal mucin expression (7 d in serum containing medium). After subtraction of the contribution of the filter and bathing medium, data were expressed as Ohms*cm^2^ of growth area.

#### Quantitative real-time PCR

Real-time RT-qPCR using TaqMan chemistry and pre-validated primers and probes (Applied Biosystems) were used to quantitate message levels of the epithelial tight junction components, Zonula occludens 1 (ZO-1) and occludin. One µg of total RNA from each cell line was reverse transcribed using iScript cDNA Synthesis Kit (Bio-Rad). Data were expressed relative to HCLE NT control cells after normalization to GAPDH (endogenous control) as described [16], [28].

### Immunofluorescence localization studies

Cultures grown for optimal mucin expression on Lab Tek chamber glass slides (Nunc) were fixed in 2% paraformaldehyde in PBS and labeled with antibodies specific for MUC1 (214D4; Upstate) and MUC16 (M11, NeoMarkers; OC125, DAKO) as previously described [13], [29].

Tight junctions on apical cells of the cultures were localized using modification of a previously described method [30]. Briefly, cultures were labeled with an antibody specific for ZO-1 (Invitrogen) or occludin (Invitrogen) following fixation in ice-cold methanol and permeabilization (ZO-1 only) with 0.02% Tween 20 in PBS. Cell surface area, expressed as pixels^2^, was measured in ZO-1 labeled images in Adobe Photoshop using the histogram function. Images were photographed at room temperature with a 25X objective on a Zeiss Photoscope III with the FITC filter with a Spot Insight camera.

For double label of tight junctions and the actin cytoskeleton, cells grown on glass chambered slides were fixed for 10 min in 2% paraformaldehyde at RT, washed with PBS, permeabilized with 1% Triton-X-100 in PBS, washed with PBS, blocked with PBS containing 1% BSA and incubated in Rhodamine-conjugated Phalloidin (Molecular Probes) for 1 h at RT. Cultures were then washed in PBS, repermeabilized, washed in PBS, reblocked as above and incubated overnight at 4°C with an antibody specific for occludin (Invitrogen). Cultures were washed with PBS, reblocked and incubated with FITC-conjugated anti-mouse IgG (Jackson Immunoresearch). Following final washes in PBS, slides were mounted with Vectashield mounting medium (Vector Labs) and photographed at RT on a Leica TCS SP5 Confocal Laser Scanning microscope (Leica).

For comparing amount of MUC16 antibody binding on apical surfaces of epithelial cells in culture to that of native tissue, discarded full-thickness human corneal epithelial sheets, removed by epikeratome for corrective refractive surgery (kindly provided by Ula Jurkunas, MD), were fixed in 4% paraformaldehyde, and MUC16 was immunolocalized on the apical cell surface using either the H185 antibody, which is specific to O-acetylated sialic acid residues on MUC16 [31], [32] or antibody CA125, Clone M11 (NeoMarkers). Cell surface area and amount of MUC16 binding were measured in Photoshop using the histogram function. Spearman Rank Correlation analyses (Instat 3 Statistical Software) were performed for the amount of antibody binding versus cell surface area.

### Immunoelectron microscopy

Cultures grown on glass chamber slides were fixed in 2% paraformaldehyde, rinsed in PBS, permeabilized with 0.3% Triton-X-100 in PBS, briefly rinsed in PBS and washed at RT with wash buffer (PBS containing 0.8% BSA and 0.1% Fish Gelatin). Cultures were then incubated in blocking buffer (PBS containing 0.8% BSA, 0.1% Fish Gelatin and 5% normal donkey serum). After a brief incubation in wash buffer, cultures were incubated overnight at 4°C in antibodies to MUC1 (214D4) or MUC16 (Clone OC125, Dako) diluted in incubation buffer (wash buffer plus 1% normal donkey serum). After extensive washes in wash buffer, cultures were incubated in 10 nm gold conjugated anti-mouse IgG (Sigma) in incubation buffer overnight at 4°C. Specimens were washed in wash buffer followed by PBS, post-fixed in ½ strength Karnovsky’s fixative, scraped off the slide in a jelly roll fashion and processed for transmission electron microscopy. Thin sections were imaged at 10,400 X on a Philips 300 transmission electron microscope (Philips).

### Scanning electron microscopy

Cell cultures were grown on 12 mm diameter glass coverslips, fixed in ½ strength Karnovsky’s fixative, dehydrated through an ethanol series, critical point dried with a SamDri-795 critical point dryer (Tousimis) and coated with chromium using an Ion Beam Coater 610 (Gatan). Samples were photographed on a JEOL 7401F Field Emission Scanning Electron Microscope (JEOL).

### Statistical analyses

Statistical analyses were performed using the Mann-Whitney U Test (for Western blots and rose bengal dye penetrance), one-way ANOVA with Student-Newman-Keuls Multiple Comparisons post-hoc test (for bacterial invasion), or Kruskal-Wallis test with Dann’s Multiple Comparisons post-hoc test (for TER and apical cell surface area) using Instat 3 statistical software or two-tailed Student’s t-test (for bacterial adherence and qPCR) using Microsoft Excel for Mac 2011 v. 14.3.5. Results are expressed as mean +/− SEM. p<0.01 was considered significant.

## Results

### Development of an assay to compare functions of MAMs in an epithelial model system

**Figure 2:**
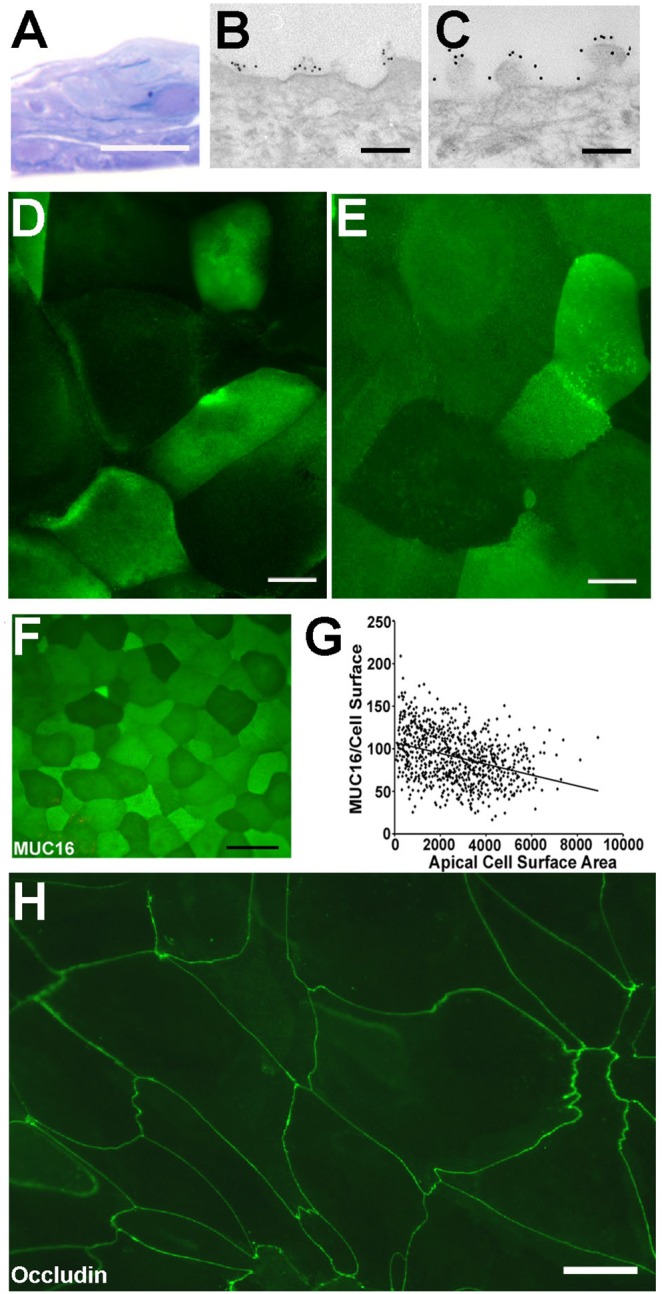
Characteristics of the epithelial cell culture model used for assay of MUC1 and MUC16 in barrier function. (A) Epithelial (HCLE) cells stratify in culture when grown for 7 d post confluence in the presence of serum. Immunoelectronmicroscopy using gold conjugated secondary antibodies that recognize anti-MUC antibodies, demonstrates the insertion of both MUC1 (B) and MUC16 (C) on the apical cell membranes of the microplicae of the cultured epithelial cells. *En face* images of nonpermeabilized epithelial cells immunolabeled with FITC conjugated secondary antibodies that bind to antibodies for MUC1 (D) or MUC16 (E) illustrate that the mucins are present on apical surfaces of cells, with some cells showing greater antibody binding than others. This feature mimics that seen in binding of MUC16 antibodies to apical cells of the native corneal epithelium (F) (G) Scatter plot of the amount of MUC16 per cell (based on H185 antibody binding intensity) and apical cell surface area illustrates the inverse correlation of surface amount of MUC16 and cell size. Spearman Rank Correlation: r = −0.36, p<0.0001. Immunolocalization of the tight junction protein occludin (H) demonstrates the presence of the tight junctions around the lateral membranes of the apical cells of HCLE cultures. Scale Bars = 20 µm in A, D, E, F, H and 0.2 µm in B, C.

A human corneal epithelial cell line was used to develop the model system to compare the functions of MUC1 and MUC16 in a mucosal epithelium. We have previously described the development and characterization of the immortalized human corneal-limbal epithelial (HCLE) cell line that differentiates to express the MUC1 and MUC16 mucin repertoire of the native epithelium [16]. These HCLE cells, when grown to confluence in serum-free medium followed by culture in serum-containing medium for 7 d, stratify to 3–5 cell layers (Fig. 2A) and have surface microplicae/microridges typical of native apical epithelial cells with MUC1 and MUC16 present on them (Fig. 2B, C). In addition, the pattern of localization of the two MAMs on the apical surface of the stratified epithelial cultures (Fig. 2D, E) is similar to that of native epithelia (Fig. 2F) in that there is a variation in the amount of the mucins on different apical cells, giving the surface a cobblestone pattern of binding. The intensity of binding of MUC16 is indirectly correlated to the apical cell surface size in native epithelia (Fig. 2G)–the cells with the largest surface area having less MUC16 antibody binding. It has been hypothesized that the largest cells are the “oldest cells” on the epithelial surface and are the cells that are about to desquamate in this stratified epithelium that turns over in 5–7 d [33]. As in native epithelia, the apical cells of the epithelial cultures form tight junctions, as demonstrated by binding of antibodies to the tight junction protein occludin along the lateral membranes of the apical cells (Fig. 2H).

**Figure 3:**
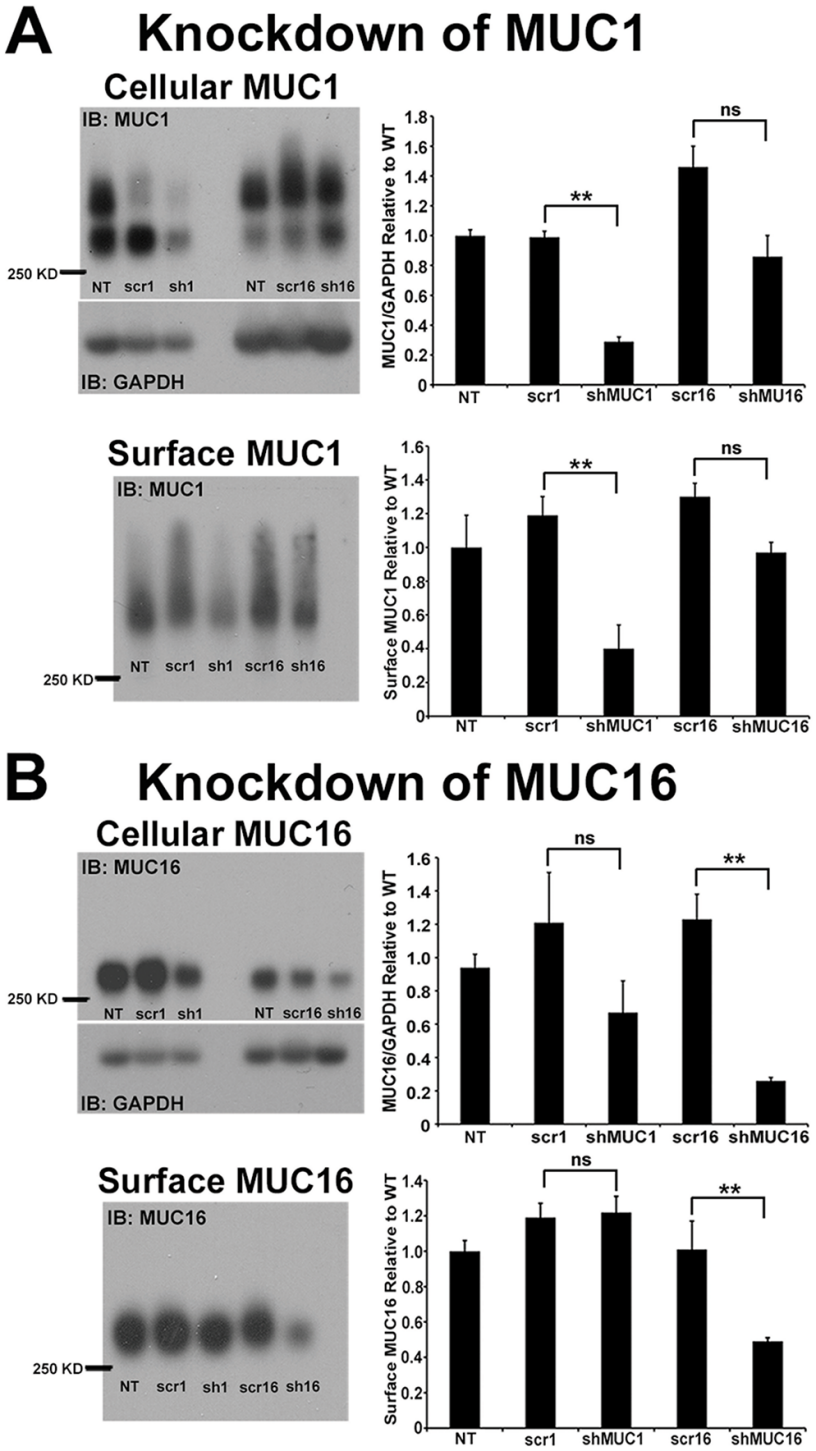
Significant knockdown of MUC1 and MUC16 proteins in both cell lysates and on apical cell surfaces following transfection with vectors expressing shMUC1 or shMUC16 sequences. (A) Western blots demonstrating that **MUC1 protein** is lower in both cell lysates (upper left) and on apical cell surfaces (lower left) of cell cultures transfected with shMUC1 containing vectors (shMUC1) compared to the non-transfected control (NT), scrambled shRNA (scr1) controls, as well as with shMUC16 containing vector (shMUC16) or its scrambled shRNA control (scr16). Alleles of MUC1 often differ in size and as they are co-dominantly expressed, two distinct protein sizes are evident on western blots. The graphs to the right of each blot, show densitometric analyses of bands demonstrating that MUC1 protein levels are significantly reduced by 71% in the cell lysates and 60% on apical surfaces relative to NT and scr1 controls and that MUC1 protein levels are not significantly reduced by knockdown of MUC16 (shMUC16) or its scrambled shRNA control (scr16). (B) Similarly, on the left are representative Western blots demonstrating that MUC16 protein levels are lower in cell lysates and biotinylated apical cell surface protein isolates of cells transfected with shMUC16 containing vectors compared to non-transfected (NT), or those transfected with scrambled shRNA for either MUC1 or MUC16 (scr1 and scr16) or shMUC1 containing vectors. The graphs on the right show densitometric analyses of blots indicating that MUC16 protein levels are significantly reduced in cell lysates by 70% and on apical surfaces by 51% in cells transfected with shMUC16 containing vectors in comparison to NT and scr16 controls. For both (A) and (B) protein samples from cell lysates were loaded based on equivalent micrograms of protein, and for cell surface proteins on equivalent cm^2^ of cell growth area. Graphic representation of the relative amounts of MUC1 (upper right) and MUC16 (lower right) was derived through densitometric analyses of the blots, cell lysates were normalized to GAPDH, and all data were expressed relative to the non-transfected control (NT). Significant if p<0.01, (**). ns = non-significant, n = 5–10.

To compare the functions of the mucins in barrier formation, HCLE cells stably knocked down for either MUC1 or MUC16 [13], using shRNA interference, were used (see Methods Section). In the shMUC1 knockdown cells, developed for this study, MUC1 protein in cell lysates was significantly reduced, by 71% (p<0.01), after transfection with two rounds of 1 µg of the psiRNA-H1b-MUC1 plasmid (InvivoGen) compared to the non-transfected control (NT) (Fig. 3A). MUC1 protein levels in the control cell lines with scrambled shRNA for MUC1 (scr1) and MUC16 (scr16), as well as in the cell line knocked down for MUC16 (shMUC16) (Fig. 3A), were not significantly reduced from the non-transfected control (p<0.01). Most importantly, in addition to the assay of the level of knockdown of MUC1 in cell lysates, the amount of the mucin present on the apical membranes of the stratified, differentiated cultures of HCLE cells was assayed by cell surface biotin labeling and subsequent capture of labeled MUC1 with immobilized avidin. Western blot analysis of the captured surface proteins revealed that the MUC1 present on the apical surface of the HCLE shMUC1 cells (Fig. 3A) was reduced by 60% compared to non-transfected control cells, and was significantly lower than all other control cell lines as well as the shMUC16 knockdown cell line (p<0.01) (Fig. 3A).

We have previously reported development of a stable knockdown of MUC16 in HCLE cells using RNA interference. The present study used the cells transduced with retrovirus containing sequence #2 MUC16 shRNA from the earlier report [13], since it gave the highest knockdown of MUC16 protein after selection with 2.5-µg/ml puromycin. In this study, MUC16 was knocked down by 74% in cell lysates compared to the non-transfected control cell line, and was significantly reduced compared to the non-transfected control and scrambled shRNA control cell lines as well as the shMUC1 cell line (p<0.01) (Fig. 3B). Most importantly, MUC16 on the apical surface of the HCLE shMUC16 (Fig. 3B) cells was reduced by 51% as compared to non-transfected control, and was also significantly lower than scrambled shRNA control cell lines and the shMUC1 cell line (p<0.01) (Fig. 3B). The data on both knockdown cell lines demonstrate that there was no significant reduction of surface MUC16 in the HCLE shMUC1 cells, nor was there a reduction of MUC1 on the surface of HCLE shMUC16 cells (Fig. 3A, B).

Tests of barrier function applied to the HCLE cells with and without knockdown of either MUC1 or MUC16 included penetration of rose bengal dye, bacterial adherence, bacterial invasion, apical tight junction formation and function, and apical cell surface area. All of these assays demonstrated distinct differences between the MAMs, MUC16 contributed to mucosal epithelial barrier function, whereas MUC1 did not.

### MUC16, but not MUC1, is a barrier to dye penetrance

**Figure 4:**
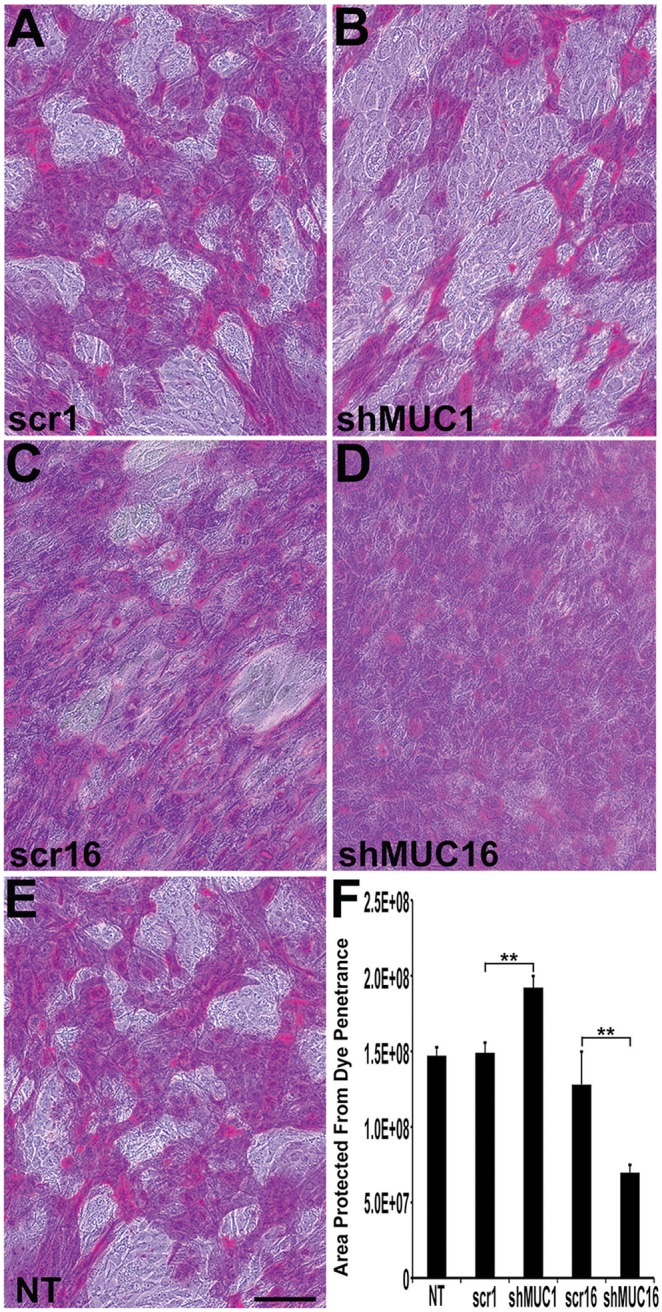
Knockdown of MUC16 enhances dye penetrance compared to knockdown of MUC1. Representative images of cultures of human corneal epithelial cells stably transfected with (A) scrambled shRNA for MUC1 (scr1), (B) shRNA for MUC1 (shMUC1), (C) scrambled shRNA for MUC16 (scr16), (D) shRNA for MUC16 (shMUC16) or the non-transfected control (NT) (E) and then incubated with rose bengal dye to determine the area of the culture that is protected from dye penetrance, an indication of a functional apical glycocalyx barrier. Rose bengal dye is excluded from islands of cells in cultures of non-transfected (NT) and scrambled shRNA controls (scr1, scr16), as well as the MUC1 knockdown cells (shMUC1) cultures. Cells knocked down for MUC16 (shMUC16) do not show as many islands of dye exclusion, indicating increased penetrance of the dye. (F) Quantitative image analyses of the area protected from dye penetrance in each cell type demonstrate a significant decrease in area protected from dye penetrance in the MUC16 knockdown cells. Conversely, there is a significant increase in the area protected from dye penetrance in the MUC1 knockdown (shMUC1) cells. Scale bar = 50 µm. **p<0.01, n = 8.

Rose bengal is an anionic dye that is frequently used to examine the integrity of the ocular surface epithelium, as binding of this dye is indicative of loss of the apical surface barrier [15]. It was previously shown that differentiated cultures of HCLE cells display islands of cells that prevent penetrance of rose bengal dye [29] and that the area of these islands is significantly decreased in the HCLE shMUC16 cells [13]. The published result was confirmed in the HCLE shMUC16 cells used in the present study and the data, when compared to islands of dye exclusion by shMUC1 and control cell lines (Fig. 4), were significantly reduced (p<0.01). Interestingly, the knockdown of MUC1 yielded the opposite result of the shMUC16 cells (Fig. 4B, F). The area of the islands of cells within the cultures that prevented dye penetrance was significantly increased in shMUC1 cells compared to the control cell lines and shMUC16 cells (p<0.01).

### MUC16 is a barrier to bacterial adherence and invasion

**Figure 5:**
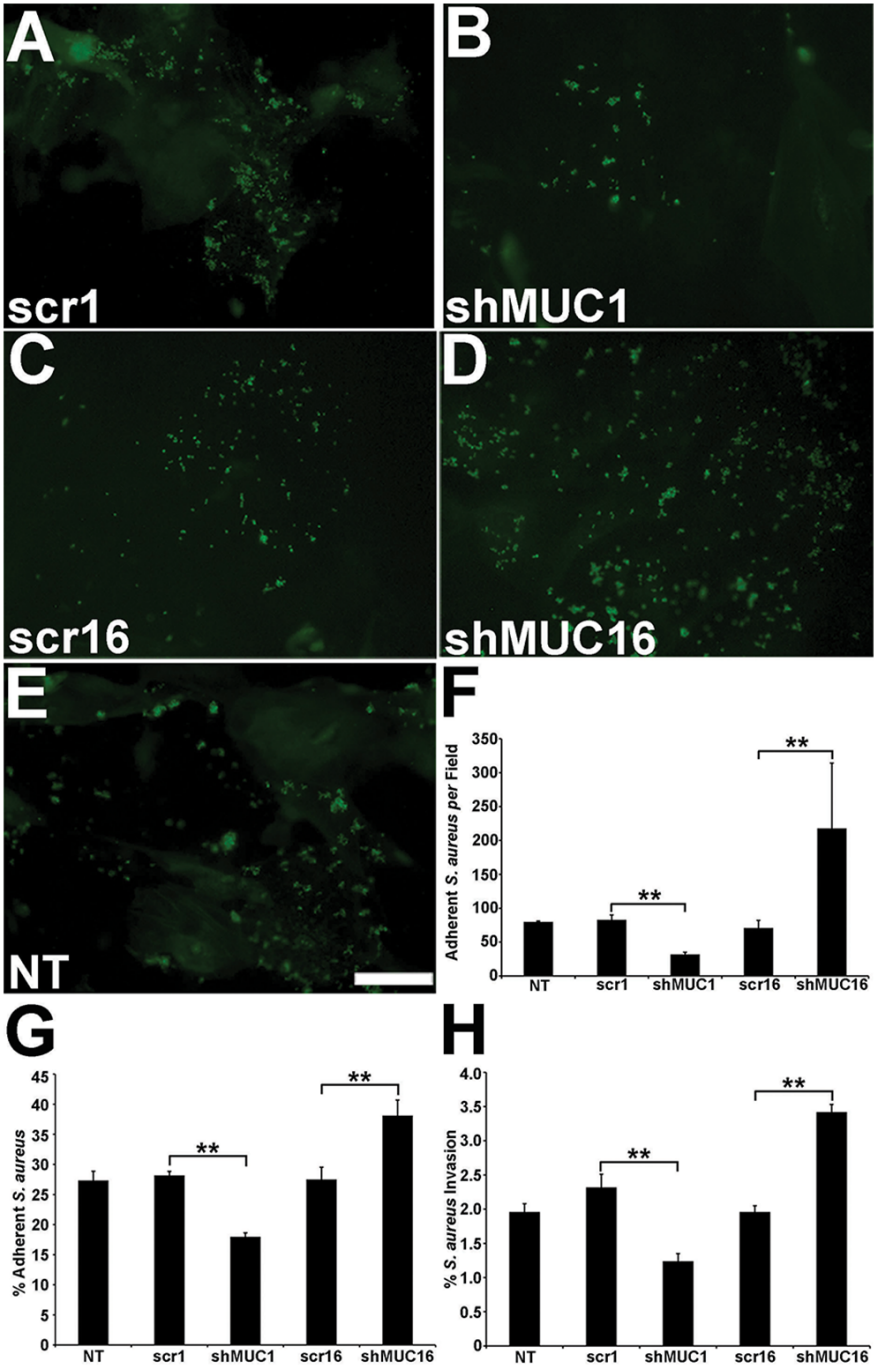
Knockdown of MUC16 increases bacterial adherence and invasion compared to knockdown of MUC1. Three types of experiments demonstrate that knockdown of MUC16 increases bacterial adherence and invasion compared to controls, and that knockdown of MUC1 enhances the barrier to bacterial adherence and invasion. In the first experiment, epithelial cultures were incubated with FITC-labeled *S. aureus* and number of adherent bacteria were counted in ImageJ. Representative images of *S. aureus* adherent to cultures are shown in (A) scrambled MUC1 shRNA control (scr1), (B) MUC1 knockdown (shMUC1), (C) scrambled shMUC16 control (scr16), (D) MUC16 knockdown (shMUC16) and (E) non-transfected control (NT). Note abundance of adherent bacteria in the MUC16 knockdown cells in image D. (F) Graph illustrating the number of FITC-labeled bacteria adherent to the epithelial cell cultures. Note that knockdown of MUC16 significantly increases adherence of *S aureus,* whereas knockdown of MUC1 significantly decreases bacterial adherence. In a second type of experiment, (G) the differences in bacterial adherence between the HCLE shMUC1 and HCLEshMUC16 and control cells were corroborated through enumeration of colony-forming units (cfus) of live bacteria recovered after the 1-h incubation with *S. aureus.* In a third experiment, (H) number of intracellular *S. aureus* that invaded the cell cultures were counted after incubation of the cultures for 4 h and determining cfus of live bacteria recovered from cell lysates following antibiotic treatment to kill surface bacteria. The three different assays demonstrate that knockdown of MUC16 is associated with a significant increase in bacterial adherence and invasion, and that knockdown of MUC1 does not increase bacterial adherence or invasion, rather the barrier to bacteria is increased. Scale bar = 30 µm. **p<0.01. n = 6–8.

It is well established that bacteria do not adhere to or invade the surface epithelial cells if the glycocalyx forming the apical surface barrier is intact [14], [34], [35]. To compare the function of MUC1 and -16 as barriers to pathogen adherence, adherence of *Staphylococcus aureus* to apical cells of the cultures of cell lines knocked down for MUC1 or MUC16, as well as control cell lines, were examined by two methods after a 1-hour incubation of cells with bacteria; first by direct visualization and quantitation of adherent FITC-labeled *Staphylococcus aureus* (Fig. 5A–F) and second, by enumerating the number of adherent live bacteria recovered following plating of epithelial cells on agar plates (Fig. 5G). Both methods demonstrated that significantly more bacteria adhered to the shMUC16 cells compared to control and shMUC1 cells. Interestingly, significantly fewer bacteria adhered to the shMUC1 cells than to the non-transfected control cells (p<0.01), suggesting that without MUC1, barrier function to pathogen adherence is improved. The increase of adherence of *Staphylococcus aureus* after MUC16 knockdown confirms our previous result with the HCLE shMUC16 cells [13].

To determine if changes in bacterial adherence translated into changes in bacterial invasion (a clearer indication of infection), the incubation of *Staphylococcus aureus* and epithelial cells was increased to 4 h, and the number of internalized bacteria was assessed using an antibiotic protection assay [14], [25]. This assay mirrored the adherence assays (Fig. 5F, G) in that the cells knocked down for MUC16 had significantly higher invasion of bacteria (p<0.01) than did the control cell lines and the shMUC1 cells (Fig. 5H). Similarly the MUC1 knockdown cultures had significantly lower bacterial invasion than did the control and shMUC16 cultures (p<0.01), paralleling the bacterial adherence assays (Fig. 5F, G). These data, as with the dye penetrance studies, demonstrate that MUC16 contributes to the glycocalyx barrier, whereas loss of MUC1 improves barrier function, perhaps by providing a more homogeneous MUC16 coverage to the apical cells.

### MUC16 contributes to TER and tight junction formation

**Figure 6:**
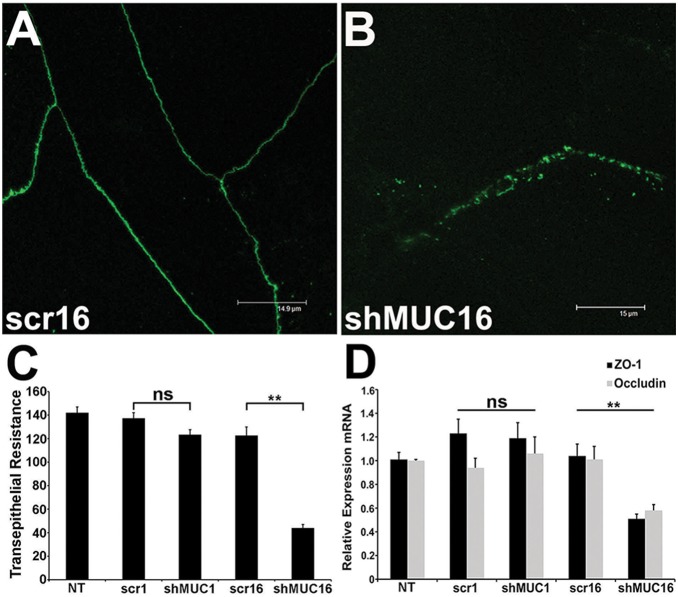
Knockdown of MUC16 results in decreased tight junction function and ZO-1/occludin expression, whereas knockdown of MUC1 has no effect on tight junctions. (A) Immunofluorescence analysis of occludin localization demonstrated normal linear distribution of occludin in the MUC16 scrambled control (scr16) cells (A) as compared to the disrupted localization seen in the shMUC16 cells (B). (C) A highly significant decrease in transepithelial electrical resistance (TER) was observed in the MUC16 knockdown (shMUC16) cell cultures compared to control cultures and shMUC1 cultures. No difference was seen in TER in the MUC1 knockdown (shMUC1) cells n = 15–30. (D) Analysis of the relative mRNA expression of two tight junction genes (ZO-1, occludin) by qPCR demonstrated a significant reduction in their message in the shMUC16 cells compared to the non-transfected (NT), or scrambled shRNA controls (scr1, scr16) and shMUC1 cells. n = 7, **p<0.01, ns = not significant.

**Figure 7:**
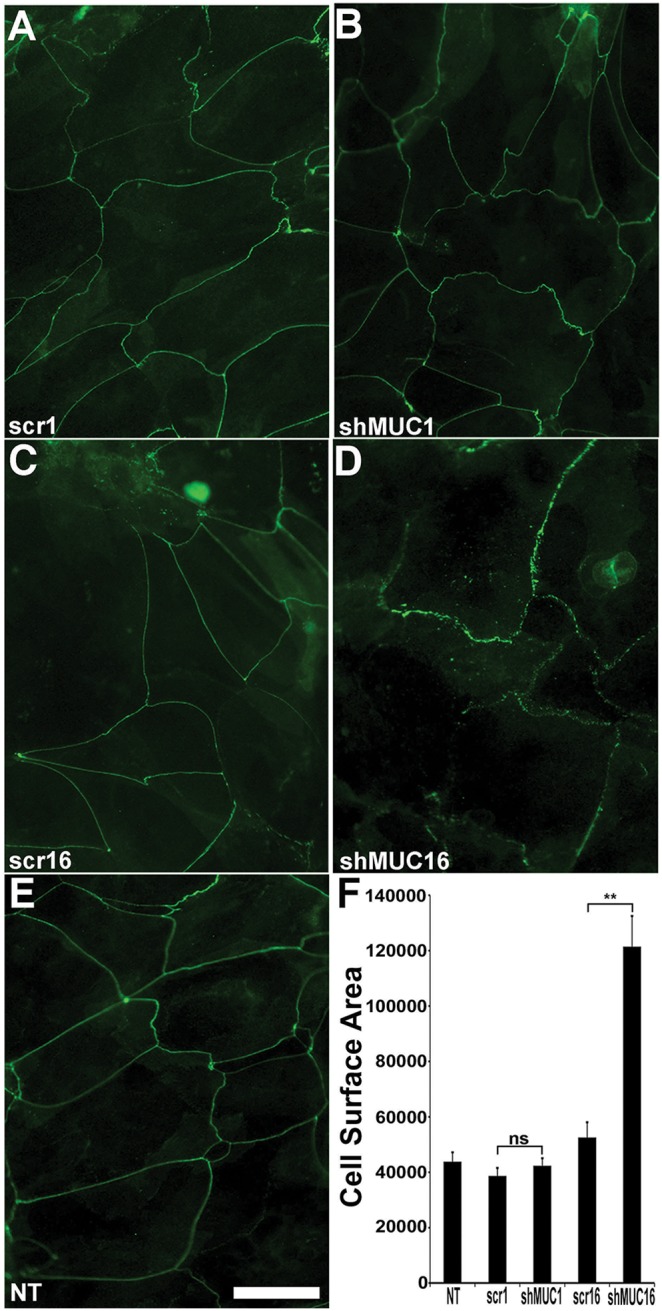
Knockdown of MUC16 results in an increase in apical cell surface area compared to knockdown of MUC1. Cell perimeters were labeled with antibodies to occludin followed by labeling with FITC conjugated secondary antibodies (A–E). Note the disruptions in the linear localization around the cell peripheries in the MUC16 knockdown cells, shMUC16 (D) compared to the continuous linear localization in the scrambled shRNA controls scr1 (A), scr16 (C) and non-transfected NT (E) controls as well as the MUC1 knockdown shMUC1 cells (B). (F) Measurement of apical cell surface area in the ZO-1 labeled cultures revealed that the mean apical surface area of the shMUC16 cells is significantly larger than those of the NT, scr1, scr16 and shMUC1 cells, all of which have comparable apical cell surface areas. Scale bar = 30 µm. **p<0.01, ns = not significant, n = 7, 5 images/sample.

We observed that MUC16 knockdown altered the continuity of Zonula occludens-1 (ZO-1) and occludin localization along lateral borders of apical cells in the epithelial cultures. In scr16 control cultures, occludin was present along the apical cell borders in a linear, undisrupted pattern (Fig. 6A), whereas in the MUC16 knockdown cells, occludin antibody binding was disrupted (Fig. 6B). Thus, we assessed TER to test tight junction function. Expression of ZO-1 and occludin RNA was also assayed. Knockdown of MUC1 in the HCLE cells did not result in a significant change in TER as compared to the control cells, but knockdown of MUC16 resulted in a highly significant decrease in TER compared to all other cell lines (Fig. 6C) (p<0.01). Assay of mRNA in the different cell types revealed no significant change in ZO-1 or occludin message in the shMUC1 cells compared to the control cells (Fig. 6D), but a significant decrease in both ZO-1 and occludin was observed in the shMUC16 cells compared to all other cells (p<0.01). ZO-1 localization mirrored that seen for occludin (Fig. 7).

**Figure 8:**
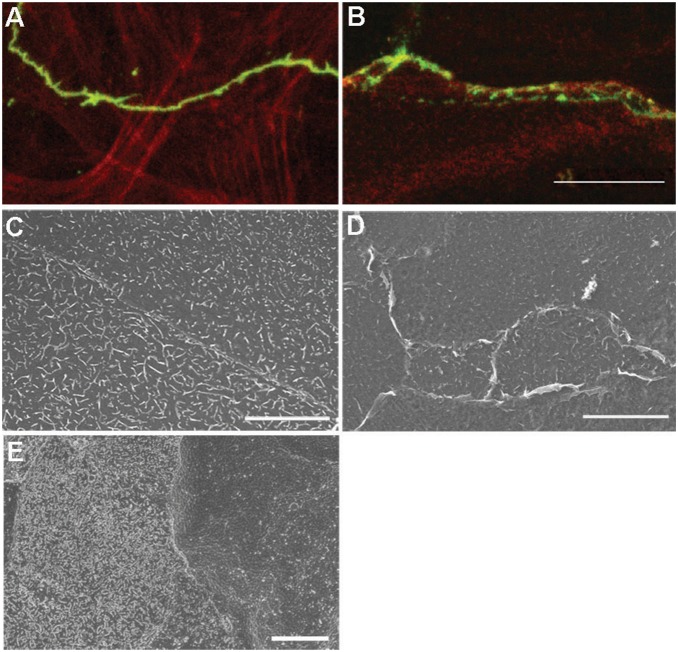
Knockdown of MUC16 results in disruption of the actin cytoskeleton associated with tight junctions and reduces surface microplicae. Epithelial cultures of non-transfected controls (A) and those transfected with shMUC16 (B) were double labeled with an antibody to occludin (green) and Phalloidin (red) to localize filamentous actin; note the actin filaments associated with the linear occludin antibody binding in A, and the lack of filamentous actin along the disrupted occludin antibody binding in B. Scanning electron micrographs of control epithelial cultures (C), shMUC16 cultures (D) and native epithelium (E). Note fewer prominent microplicae in the cells knocked down for MUC16 in D and also in the larger darker cell of native epithelium (E), that were shown (Fig. 2F) to bind less antibody to MUC16. The larger darker cells show fewer microplicae than neighboring smaller light cells. Scale bars = 15 µm in A, B, 10 µm in C, D, 5 µm in E.

Since the cytoplasmic tail of MUC16 has an ezrin, radixin, moesin, (ERM) binding domain that allows the ERMs to link to filamentous actin [13], it is possible that the lack of MUC16 results in alteration of an apical actin cytoskeleton cortical mat and terminal web formation, which would influence tight junction formation [36]. In fact, comparison of non-transfected control and shMUC16 epithelial cultures double labeled with antibodies to occludin to delineate tight junctions, and phalloidin to localize filamentous actin, demonstrates that filamentous actin association to lateral membranes is disrupted in the shMUC16 cells (Fig. 8A, B).

### MUC16 influences apical membrane surface area

Linkage of membrane-spanning proteins to the actin cytoskeleton by members of the ERM family of proteins is known to regulate development of cell membrane surface projections, in that, overexpression of the ERMs drives increased length of microvilli [37], [38]. Given that the cytoplasmic tail of MUC16 can associate with the actin cytoskeleton via its ERM-binding domain [13] and that MUC1 has been shown in breast cancer cells to mediate actin cytoskeletal membrane protrusive motility by way of ICAM-1 ligation and an Src signaling cascade [39], we hypothesized that MUC1 and MUC16 may, through cytoskeleton association, induce membrane folds or projections on the epithelial cells’ apical surface. Lack of such associations could, we hypothesized, lead to increased apical cell surface area. To test the hypothesis, lateral cell membranes of the apical cells of stratified, differentiated cultures of non-transfected and scrambled shRNA controls, and shMUC1 and shMUC16 cells were delineated with tight junction markers (ZO-1 and occludin) (Fig. 7A–7E) and surface area of the apical cells was measured using the ZO-1 images (Fig. 7F). Measurement of the apical surface area of the knockdown and control cell types (Fig. 7F) revealed that the shMUC16 cells had a significantly larger surface area than did the control and shMUC1 cells (p<0.01). There was no significant difference in apical cell surface area between the shMUC1 cells and control cells (Fig. 7F). These *in vitro* data showing that MUC16 on apical cells was related to cell surface size correlated to that shown earlier on native corneal epithelium (Fig. 2F, G). In native epithelium, there was a highly significant inverse correlation between the intensity of binding of antibodies to MUC16 and apical cell surface area. (MUC16: Spearman rank correlation r value = −0.36,; p<0.0001).

Comparison of scanning electron micrographs of the surfaces of the epithelial cultures of non-transfected controls and shMUC16 cells suggested that fewer microplicae are present on the shMUC16 cells (Fig. 8C, D). However, as we reported previously [13], microplicae do occur in the knockdown cells, perhaps due to remnant MUC16 as surface MUC16 was knocked down by 51%, other membrane molecules that associate with ERMs to induce microplicae, and/or artifact due to critical point drying. As in native epithelium, the larger cells at the surface of the stratified epithelial cultures, which showed less binding of MUC16 antibodies appeared also to have fewer, more sparsely distributed microplicae (microridges) (Fig. 8E). Others have noted that fewer microplicae are present on larger cells at the corneal surface [40]. Diminution in microridges on the surface of the shMUC16 cells may have resulted in the larger surface areas of both the shMUC16 cells and the large native apical epithelial cells, each of which bound fewer MUC16 antibodies.

These data provide evidence that apical cell surface area increases when less MUC16 is present on the apical cell surface. Perhaps, as cells age at the epithelial surface, shedding of the MUC16 ectodomain, which is known to occur constitutively in vitro [41] and in vivo [42], causes loss of association to the actin cytoskeleton and loosening of the lateral adherens and tight junctions to allow desquamation.

## Discussion

Taken together, the data presented herein demonstrate distinct differences in the contributions of MUC1 and MUC16 to mucosal epithelial barrier function when present in the same epithelial apical membrane. Knockdown of MUC16 demonstrated that the MAM is a barrier to dye penetrance, bacterial adherence and invasion, is involved in tight junction function and formation, and apical cell surface area. On the other hand, knockdown of MUC1 showed that this MAM did not contribute to the barrier to dye penetrance and bacterial adherence nor did it to tight junction formation and TER, or to cell surface area. Indeed, surprisingly, for several of these barrier functions, knockdown of MUC1 did just the opposite–the barrier to dye penetrance and bacterial adherence and invasion was enhanced in cells with less MUC1. Perhaps in those epithelia that express these two mucins, MUC16, through its extraordinary large size, which is approximately 20 times that of MUC1, along with its heavy O-glycosylation, provides the major barrier. The fact that the loss of MUC1 allows an even more effective barrier, may be a result of a more homogeneous MUC16 rich glycocalyx.

The mechanism by which MUC16 provides an especially robust barrier may be due, not only to its exceptional ectodomain length of approximately 22,000 amino acids, which has been estimated to extend 250–300 nm from the cell surface [43] but also to its N-terminal half, which is heavily O-glycosylated. Inhibition of MUC16 O-glycosylation by knockdown of T-synthase, a galactosyltransferase required for synthesis of core1 O-glycans, resulted in decreased surface O-glycosylation and increased dye penetrance, indicating the importance of O-glycan in barrier function of the MAM [15]. Furthermore, the multivalent carbohydrate binding lectin galectin 3 binds to the glycans of MUC16 (as well as MUC1), and disruption of the galectin 3-O-glycan interaction with competitive carbohydrate inhibitors results in dye penetrance, and abrogation of barrier function [15]. Thus, the molecular mechanism of MAM barrier function is that of extended, heavily glycosylated MAM ectodomains, linked to one another through multimeric galectins. A longer molecule, such as MUC16, would provide more surface for glycan-galectin interactions to hold the molecules in a tight barrier conformation. In a glycocalyx in which the MAM repertoire is mixed, several levels of MAM-galectin association may be present with MUC16 ectodomains extending further from the cell membrane than MUC1. This could provide an uneven, mixed-length extension of the MAMs in the glycocalyx, thus providing differences in length that pathogens and dyes must traverse to reach the cell surface. Abrogation of expression levels of MUC1 with its shorter ectodomain, leaves a more uniform MUC16, glycan-rich, uniform barrier with a more robust barrier function.

The data indicating that decrease in expression of MUC1 enhances barrier function in the corneal epithelium seems to contradict the studies cited in the introduction to this manuscript that demonstrate that MUC1 prevents pathogen adherence and penetrance. While our study demonstrates a greater role for MUC16 in barrier function, it does not eliminate the possibility that MUC1 has a barrier role in other epithelia, especially in those epithelia that do not express the very large mucin MUC16. The data do suggest, however, that barrier functions of each of the MAMs expressed by a mucosal epithelium may need to be evaluated in the context of the MAM repertoire of that epithelium. In fact, in a study of the role of MUC1 in adenoviral access to the respiratory epithelium, the authors state that “the inability to achieve high gene transfer efficiency, even in mice with a depletion of Muc1, suggested that other glycocalyx components, possibly other tethered mucin types, also provide significant barrier to AdV”[9].

It would be ideal to verify the data on the functions of the human mucins MUC1 and MUC16, provided herein, in mice null for the human homologues designated Muc1 or Muc16. Indeed such animals have been produced [11], [44], [45]. There are however major differences in the structures of the human and mouse MAMs, particularly in their ectodomains sizes and homologies. The ectodomain of human MUC1 is 1140 AA’s whereas in the mouse homologue, it is approximately 550 AA’s. The C terminal cytoplasmic tail region of the MUC1 homologues is 72 AA and is the most homologous region of the molecule. Similarly, the ectodomain of human MUC16, is approximately 22,110 AA’s, whereas the mouse Muc16 is much smaller at approximately 8,830 AA’s. As with MUC1 the cytoplasmic tail sequence of MUC16 is conserved between species and is 35AA’s in length. Most importantly however, in terms of comparing the functions of the two mucins, the mucosal epithelial expression pattern of MUC16 is very different between the two species. In human’s MUC16 is expressed in corneal and uterine epithelial surfaces whereas in mice it is not [11]. These differences, plus variations in mucin glycosylation characteristics that exist between species, make comparisons between specific mucin functions across the two species difficult.

To our knowledge the only other study in which the comparative barrier function of two MAMs has been tested has been in the role of MUC16 in trophoblast adherence in the human endometrium [5]. Data from that study agree with that of the present study in that MUC16 was shown to be a barrier to trophoblast cell adherence, whereas MUC1 was not. These studies initiated from the demonstration that MUC16 was dramatically shed from the apical glycocalyx of endometrial epithelium, 5–7 days after LH surge, the time of trophoblast adherence to the endometrium, which initiates implantation. Immunohistochemical analysis of the endometrial surface indicated that both MUC1 and MUC16 are expressed by the endometrium. MUC1 was not, however, shed from the ciliated cells at LH 5–7. MUC16 and MUC1 were independently and stably knocked down in an endometrial cell line using shRNA methods and then tested for adherence of cells from a trophoblast cell line. Trophoblast cells adhered in greater numbers to the cells knocked down for MUC16, whereas knockdown of MUC1 did not affect adherence.

In addition to its expression by the corneal epithelium, the large MUC16 MAM is expressed by tracheal-bronchial epithelia, and endometrial and cervical epithelia [5], [44], [46], [47].

All these epithelia, except for the corneal epithelium, express MUC4 in addition to MUC1 and MUC16, but like MUC1, MUC4 is a much smaller mucin than MUC16. Perhaps the mucosal epithelia where MUC16 is expressed, need an especially robust glycocalyx to provide a barrier to pathogen and cell adherence. The ocular surface and respiratory epithelia are directly exposed to environmental particulates and pathogens and, thus, require an exceptionally efficient and robust barrier. The female endometrial and cervical epithelia need a surface that will prevent pathogen and cell-cell adherence, particularly in relation to sperm and unfertilized ova. The gastrointestinal epithelial surfaces, although exposed to large numbers of bacteria, do not express the large MUC16. Goblet cells within these epithelia do however secrete large amounts of mucins in which pathogens are trapped, and upon which pathogens feed.

A surprising finding of this study was that knockdown of MUC16, but not MUC1, disrupted tight junction formation and resultant tight junction function as measured by TER. The knockdown also showed downregulation of ZO-1 and occludin expression, and apical cells of the stratified cell cultures show larger apical surface area (Figs. 6 and 7). MUC16 has a polybasic juxtamembrane amino acid domain, RRRKK, in its cytoplasmic tail sequence, and we have shown previously, that synthetic peptides mimicking the MUC16 CT, bind the actin cytoskeleton linker moesin, a member of the ezrin-radixin-moesin (ERM) family of proteins [13]. MUC1 has an RRK sequence near the transmembrane domain; however, peptides mimicking the MUC1 cytoplasmic tail domain, do not, in our hands, bind ERM proteins. These data indicate that the MUC16 cytoplasmic tail can, through ERM binding, link the cytoplasmic tail of MUC16 to filamentous actin. ERMs, by linking membrane-tethered proteins to filamentous actin are known to be involved in development and lengthening of cell surface membrane protrusions such as microvilli. They are also known to influence adherence junction formation (for review see [48]). Perhaps knockdown of MUC16 and, thus, loss of cytoplasmic tail association to ERM’s in apical membranes of corneal epithelia, results in the lack of association of the membrane to the actin filaments that insert into surface microplicae (microridges) (Fig. 1A) and to the apical actin network involved in cell surface membrane organization, leading to the increase in cell surface area demonstrated in Figure 7. Concomitantly, the apical cortical web of actin filaments may not form, leading to the disrupted adherence and tight junction formation (for review of the linkage of adherens and tight junction formation see [36]) and, thus, the lowered TER observed in this study. The data showing the role of MUC16 in tight junction formation suggests an important role for the MUC16 cytoplasmic tail in maintenance of epithelial barrier function through anchorage of MUC16 in its position at the apical surface to the actin cytoskeleton. To our knowledge this is the first demonstration of the association of a MAM with tight junction formation.

The question arises, could the disruption of tight junctions rather than loss of the MUC16 ectodomain in the MUC16 knockdown cells be responsible for the increase in dye penetrance and bacterial adherence and invasion seen in this study? Our previous data indicate that the ectodomain is acting as the barrier to dye penetrance and bacterial adherence. First of all, rose bengal dye will cross the cell membrane into cells that lack a mucin surface and that have no associated tight junctions. For instance, rose bengal penetrates fibroblasts in culture, whereas those stratified epithelia expressing apical mucins, develop a barrier to dye penetrance [29]. The dye also penetrates epithelial cells in cultures that have not been induced to stratify and produce apical surface mucins, i.e., pre-confluent and confluent but not stratified cultures [13]. The dye also penetrates epithelial cells in which O-glycosylation has been molecularly blocked, but in which MUC16 is expressed. The paper describing that data, also demonstrates quite conclusively that the tight junctions and transepithelial resistance are not altered by the deglycosylation [15]. Finally, enzymatic release of the MUC16 ectodomain by *Streptoccus pneumoniae* derived zinc metalloprotease C, which also does not alter tight junctions, also increases rose bengal dye penetrance [14]. Taken together, this body of data indicate that the rose bengal dye penetrance is through apical membranes into the cytoplasm of cells that lack the MUC16 ectodomain or in which its glycosylation has been altered. A similar result was obtained regarding the role of the MUC16 ectodomain in bacterial adherence and invasion. In previous work we demonstrated that enzymatic removal of the ectodomain of MUC16 by *Streptococcus pneumoniae* derived zinc metalloproteinase C, caused, in addition to increased rose bengal dye penetrance, increased bacterial adherence and penetrance [14]. We found no loss of tight junctions or decrease in transepithelial resistance with this treatment.

All the studies reported herein show no function for MUC1 in the barrier parameters tested. If MUC1 does not contribute to the barrier functions at the surface of epithelia in which MUC16 is expressed, what is the function of the molecule? It appears clear from a series of studies, particularly in cancer cells, that the MUC1 CT participates in signaling pathways (for review see [2]). As an example, MUC1 has an EGF-like domain near the transmembrane domain, and binding of EGF receptors has been shown to phosphorylate tyrosine residues in its short cytoplasmic tail [26]. It is not clear if this activity occurs in native epithelia. Other studies have demonstrated a role for MUC1 in native immunity. For example in respiratory epithelia, MUC1 negatively regulates TLR signaling in response to infection and inflammation [49]. These non-barrier functions ascribed to MUC1 indicate the “multifunctional” properties sometimes ascribed to the mucin [2]. Perhaps when MUC1 is present on epithelia on which larger membrane mucins such as MUC16 are present, its function as a barrier is diminished, but its signaling activity in response to the external environment remains.

In summary, when barrier functions of the membrane-anchored mucins MUC1 and MUC16 are tested within the same epithelial type, MUC16, with its much larger ectodomain and actin-associated cytoplasmic tail, is the predominant contributor to the barrier against pathogen adherence/penetrance and dye penetrance, and it participates in tight junction formation.
